# Effects of Family-Centered Empowerment Model Based Education Program on Quality of Life in Methamphetamine Users and Their Families

**DOI:** 10.5812/ircmj.13375

**Published:** 2014-03-05

**Authors:** Afsaneh Ghasemi, Abbass Rahimi Foroshani, Nasrin Kheibar, Marziye Latifi, Narges Khanjani, Mohammad Eshagh Afkari, Mohammad Hossein Taghdisi, Faranak Ghasemi, Davoud Shojaeizadeh, Maryam Dastoorpour

**Affiliations:** 1 Department of Health Education and Promotion, School of Public Health, Fasa University of Medical Sciences, Fasa, IR Iran; 2 Department of Epidemiology and Biostatistics, School of Public Health, Tehran University of Medical Sciences, Tehran, IR Iran; 3 Department of Nursing, Behbahan Faculty of Medical Sciences and Health Services, Behbahan, IR Iran; 4 Department of Health Education and Promotion, School of Public Health, Tehran University of Medical Sciences, Tehran, Iran; 5 Department of Epidemiology and Biostatistics, School of Public Health, Kerman University of Medical Sciences, Kerman, IR Iran; 6 Mental Health Research Center, Iran University of Medical Sciences, Tehran, IR Iran; 7 Department of Education and Promotion, School of Public Health, Iran University of Medical Sciences, Tehran, IR Iran; 8 Shiraz University of Medical Sciences, Shiraz, IR Iran; 9 Research Center for Modeling in Health, Institute for Futures Studies in Health, Kerman University of Medical Sciences, Kerman, IR Iran

**Keywords:** Family Nursing, Social Support, Quality of Life, methamphetamine

## Abstract

**Background:**

Nowadays there are more concerns about drug treatment of methamphetamine abusers whereas quality of life (QOL) related supportive psychotherapy is less credited.

**Objectives:**

This study aimed to evaluate the effects of family-centered empowerment model on social support and QOL of methamphetamine users and their families.

**Patients and Methods:**

This study was a randomized clinical trial; individuals were randomly allocated to three groups: a group for educating methamphetamine users in recovery (95 subjects), a group for educating a family member of methamphetamine users in recovery (95 subjects) and a control group (95 subjects). Data collecting instruments were standard questionnaires of social support and health-related quality of life (HRQOL). Data were analyzed using χ2-test, t-test, paired t-test, Pearson’s correlation and ANOVA.

**Results:**

Mean scores of QOL and social support dimensions changed significantly in two intervention groups (P < 0.0001), but didn’t change in the control group (P > 0.05). Also, there was a positive significant relation (P < 0.05) between total social support and all dimensions of QOL for all study groups.

**Conclusions:**

Family-centered empowerment model, easily adapted to methamphetamine users and their families, leads to improved social supports and QOL.

## 1. Background

Methamphetamine, also known as crystal, meth, ice or glass is a group of psychoactive drugs. This highly addictive substance is increasingly available and its abuse has grown recently due to its easy manufacture in illegal laboratories (1). As reported by the National Survey on Drug Use and Health, only in the U.S. population, 5.3% (more than 12 million people) have used it at least once in their lifetime (2). Also, the latest statistics published by the United Nations Office on Drugs and Crime in 2010 indicated that methamphetamine abuse has increased seriously worldwide in the past 5 years and Iran has the fifth place in methamphetamine use ranking, after Mexico, USA, China, and Thailand (3). Methamphetamine users are a vulnerable group in the society; besides physical consequences, they face psychological, emotional, social and financial problems which affect their QOL adversely and keep them from their routine activities (4, 5). Often methamphetamine abuse leads to various problems including serious mental disorders, involvement in crimes, marital problems and divorce, socio-emotional problems and job instability, all related to QOL and mostly ignored (6). Until recently drug therapy has been considered solely, whereas QOL related supportive psychotherapy is less credited (4).

As defined by the World Health Organization, QOL is individuals’ perception of their position in life in the context of their culture and value systems, in which they live and in relation to their goals, expectations, standards and concerns (7). The concept of QOL has been a part of addiction and substance abuse literature in a variety of studies, like assessing well-being and life satisfaction in addicts (8, 9), evaluating severity and side effects of addiction and relapse (10) and also assisting medical staff to decide on a suitable treatment for addiction and substance abuse (10, 11). Research evidence indicates that addiction and substance abuse is related to lower QOL (12, 13). Social support is a predictor for QOL, especially for methamphetamine users, which can reduce symptoms of depression in addicts and be a strong incentive for them to quit and stay abstinent (14). Strong and effective social support for drug abusers, have been highly successful, in keeping away from drugs and changing attitudes towards problems, improving physical and mental health and ultimately QOL (15). Social support is important for treatment success (16). In 2010, So-Kum Tang et al. showed a statistically significant relation between desire to quit and different dimensions of social support and QOL (17). Effects of social support on substance abusers can be explained in three terms: first, social support can reduce social isolation and withdrawal; thus creating strong interpersonal relationships, second, reduced social isolation can prevent contacting other addicts and third, social support can help addicts in problem solving and anger management through communication with strong people and prevent relapse (15). It is worth noting that drug abuse, like methamphetamine addiction, affects not only the individuals but also their family and even their community. In that matter, both addicted people and their families need to be empowered for overcoming addiction, with more social support and improved QOL. Most experts believe that empowerment is a dynamic, positive (18, 19), social and interactive process (20); a process which is formed through connecting others (21) and leads to improved QOL, responsibility, better interaction with care givers, satisfaction (22), better response to treatment (23) and even preventing side effects (24). Family empowerment model is designed upon effectiveness of the individual and other family member’s role on the three motivational, psychological (self-esteem, self-control and self-efficacy) and self-problem characteristics (like perceived knowledge, attitude and perceived threat). Until recently this strategy has been designed to improve QOL in patients with chronic diseases like anemia, thalassemia, diabetes, asthma and epilepsy but to our knowledge it has not been implemented for drug addicts, especially methamphetamine users. The main goal of family empowerment model is to strengthen the family (patient and other members) in order to improve the health level. The alarmingly high prevalence of methamphetamine abuse in Iran and the world, necessitates an effective family-centered plan to control drug abuse and particularly methamphetamine use.

## 2. Objectives

This study aimed to evaluate the effects of the family-centered empowerment model on social support and QOL of methamphetamine abusers and their families.

## 3. Patients and Methods

### 3.1. Study Population and Sampling

This was a randomized clinical trial with an educational intervention and a pre-post design involving methamphetamine-dependent individuals and their families. All subjects were in recovery and were admitted to clinics of Tehran University of Medical Sciences during a 12-month period in 2012-2013. Inclusion criteria for methamphetamine-dependent individuals were: age between 20-64, no longer than 10 years of abuse history, in recovery and passed detoxification step and willingness to participate in this study. Exclusion criteria were relapse and unwillingness to participate. Subjects were selected among methamphetamine dependent patients and their family members based on random numbers from the table and then randomly allocated to two intervention groups and a control group. Type of randomization was simple. Intervention groups include: one group for educating methamphetamine users in recovery (95 subjects), one group for educating a family member of methamphetamine users in recovery (for single patients: father, mother, sister or brother and for married patients: wife or children) (95 subjects) and the control group which included methamphetamine users in recovery with no intervention (95 subjects).

### 3.2. Sample Size

In this study samples were chosen based on odds ratio and a study (25) that showed almost 50% of uneducated methamphetamine users have low QOL and the odds ratio of QOL in educated group to uneducated group is 2.5. Assuming a confidence level of 95%, a power of 80% and the following formula, the optimum sample size for each study group was 95 and a total of 285 subjects were chosen.

P_1_ = 0.5, P_2_ = (P_1_ × OR) / (1 + (OR - 1) P_1_) = 0.7, P = (P_1_ + P_2_) / 2 = 0.6, 1 - P = 0.4

n = (2 [(Z_(1-α⁄2)_ + Z_(1-β)_)]^2^ × [(P) × (1 - P)]) / [(p_1_ – p_2_)]^2^

Note that TUMS clinics are equally located in northern, southern, western and eastern parts of the city and were representative of methamphetamine abusers from different regions of Tehran, willing to quit.

### 3.3. Data Collection

Data collection instruments included: 1- demographic check list: age, gender , education level (high school degree or lower, associate degree, bachelor's degree and higher), marital status (single - married); 2- the Persian version of the Short Form Health Survey (SF-36) including 36 questions measuring eight dimensions of quality of life: physical functioning, social functioning, role limitation (physical and emotional), bodily pain, mental health, vitality and general health. Each dimension has a score of 0-100, with higher scores indicating a better health status. The reliability and validity of the Iranian version has been approved by Montazeri et al. (26). The 3-perceived social support questionnaire adapted from Canty-Mitchell et al. (27), with its reliability and validity verified by Mohammadian et al. (28) has 12 questions on a Likert scale of 7, items ranging from "strong agreement" to "strong disagreement". A higher score indicates more support from friends, family and other important people.

### 3.4. Intervention Program

The main purpose of this intervention program was improving social support, health (physical, mental and understanding social support) and quality of life in methamphetamine addicts and their families. Also, enhancing the patients’ social and psychoanalytical function, supporting self-confidence, informing them regarding the disease and its limitations, preventing reoccurrence of disease, empowering the patient against stressful situations and understanding social support and important supportive sources like family, important people in an individual's life like wife and friends.

The general principles stated in this program were educating, ensuring, guidance, empathy, encouragement and the chance to express emotions to promote social support from others. The intervention program was performed in nine sessions as follows:

1. Introducing group members, stating the purpose of applying treatment, definitions related to drugs, transfer ways and prevention,
2. Definitions of quality of life and its dimensions,
3. The importance of understanding supportive resources and optimal usage of these resources while treating addiction,
4. Training problem solving methods in order to encounter life in a sane way, seeking opportunities to express emotions to group members according to identification of social support resources,
5. Training relief techniques and positive visualization to reduce anxiety and internal tranquility,
6. Analyzing the sense of sin and alleviating it, chances to express emotions for group members,
7. Training methods of increasing confidence and self-esteem based on abilities and supporting them to do daily activities,
8. Teaching the importance of purposes and targeting methods,
9. Stating a summary of the last sessions' topics and presenting feedback by repeating emphasis statements.

### 3.5. Ethical Considerations

Ethical issues (including plagiarism, informed consent, misconduct, data fabrication and/or falsification, double publication and/or submission, redundancy, etc.) have been completely observed by the authors. The Ethics Committee of Tehran University of Medical Sciences approved the study protocol. For ethical reasons, at the end of the study the control group was also educated. Informed consent (oral and written) of all participants was obtained and the Declaration of Helsinki was followed throughout the study.

### 3.6. Statistical Analysis

The normality of data was tested and confirmed by Kolmogorov-Smirnov test. Descriptive statistics like mean and standard deviation were calculated and statistical procedures including χ2-test, t-test, paired t-test, Pearson’s correlation and ANOVA were conducted. Assumptions of homogeneity of variances were examined with Levin's test. Based on the results, assumptions of homogeneity of variances in variables total social support and total quality of life were approved in three groups under study (P > 0.05). Data was analyzed by SPSS 20.0 software. An α level less than 0.05 was considered significant.

## 4. Results

The mean age of patients and their families were 23.2 (SD = 12.8) and 31.1 (SD = 8.2), respectively. Most methamphetamine-dependent subjects in this study were 15-34 year old (69.5%) and 75.3% were males with an education level of high school or lower (63.7%). Table 1 contains a detailed summary of demographics for each study group.

**Figure 1.**
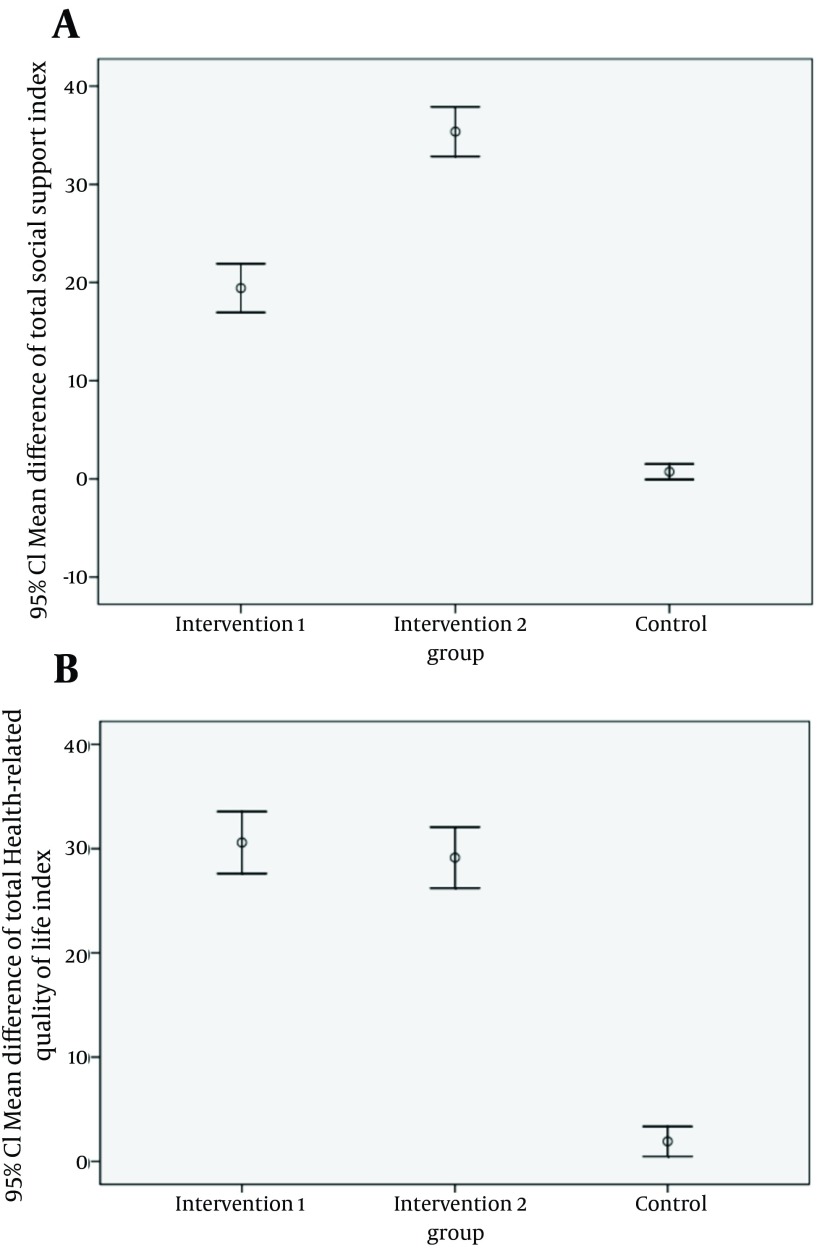
Comparing the Mean Difference of Total Social Support and Total Health-Related Quality of Life Index Scores Between the Three Groups

T-test and χ2 confirmed homogeneity of demographic variables including: age (P value = 0.89), gender (P value = 0.06) and education level (P value = 0.70), between intervention and control groups, before and after intervention 1. Independent t-test showed that mean scores of social support and QOL dimensions before intervention were not significantly different for intervention 1 and control groups (P value > 0.05) but after the intervention there was a significant difference (P value < 0.05) (Tables 1 and 2 ). Also, according to paired t-test, mean scores of social support and QOL dimensions after intervention, in intervention group 1 had significantly changed compared to before intervention (P value < 0.0001) but not in the control group who were not educated (P value > 0.05) (Tables 1 and 2). Another paired t-test indicated that mean scores of social support and QOL dimensions before and after intervention in intervention group 2 (including family members of the on rehab patient) were statistically different (P value < 0.0001) (Tables 4 and 5). In other words, it seems like the family-centered empowerment model has improved social support and QOL in intervention groups 1 and 2. Pearson correlation showed that total perceived social support is positively correlated with all dimensions of QOL in all three groups (P value < 0.05). The mean difference total social support and total QOL scores were compared between the three groups. The mean difference total social support and total QOL scores calculated by mean total social support and total QOL scores in pre intervention minus mean total social support and total QOL scores in post intervention. It was significantly greater in Intervention 1 and Intervention 2 groups than the control group. Consequently ANOVA test showed there were significant differences in mean differences of total social support and total QOL scores between the three groups (P < 0.001). These results were presented by error bar plot in Figure 1.

**Table 1.** The Distribution of Demographic Variables in the Three Groups ^a^

Variable	Intervention Group 1 (n = 95)	Intervention Group 2 (n = 95)	Control Group (n = 95)
**Age**	32.84 ± 7.9	23.2 ± 12.8	29.33 ± 8.3
**Sex**			
Male	66 (69.5)	37 (38.9 )	77 (81.1)
Female	29 (30.5)	58 (61.1)	18 (18.9)
**Education Levels**			
Under diploma and diploma	60 (63.2)	8 (8.4)	61 (64.2)
Post diploma education	13 (13.7)	54 (56.8)	16 (16.8)
Bachelor and higher	22 (23.2)	33 (34.7)	18 (18.9)

^a^Data are presented as mean ± SD or No (%)

**Table 2.** Mean Scores of Social Support Dimensions in Intervention 1 (Addicts) and Control Groups, Before and After the Educational Intervention ^a, b^

Intervention 1 and control groups	Personal			Family Support			Friend Support		
	Pretest	Post test	Paired t test	Pretest	Post test	Paired t test	Pretest	Post test	Paired t test
**Intervention group 1**	11.9 ± 6.1	17.8 ± 6.1	< 0.0001 ^c^	15.1 ± 7.6	20.7 ± 4.8	< 0.0001 ^c^	12.8 ± 6.4	18.8 ± 6.2	< 0.0001 ^c^
**Control group**	11.5 ± 5.2	11.8 ± 5.3	0.28	12.3 ± 6.6	13.6 ± 5.5	0.09	12.4 ± 5.6	12.4 ± 5.7	0.23
**t test**	0.62	< 0.0001^ c^	-	0.52	< 0.0001^ c^	-	0.68	< 0.0001 ^c^	-

^a^ The attainable score is 4-28 in all Dimensions.

^b^ Data are Presented as mean ± SD.

^c^ It is significant at α level less than 0.05.

**Table 3.** Mean Scores of Quality of Life Dimensions in Intervention 1 (Addicts) and Control Groups, Before and After the Educational Intervention ^a, b^

	Intervention Group 1	Control Group	t Test
**Physical functioning**			
Pretest	74.5 ± 21.4	72.3 ± 26	0.18
Posttest	92.3 ± 10.2	73 ± 26.1	< 0.0001
Paired t test	< 0.0001 ^c^	0.14	-
**Physical Role**			
Pretest	45± 35.7	39.2 ± 39	0.29
Posttest	99.2 ± 5.7	41.6± 0.2	< 0.0001 ^c^
Paired t test	< 0.0001 ^c^	0.06	-
**Body pain**			
Pretest	67.8 ± 20.4	72 ± 26.9	0.23
Posttest	95 ± 8.6	73.3 ± 6.8	< 0.0001 ^c^
Paired t test	< 0.0001 ^c^	0.16	-
**Vitality**			
Pretest	57.4 ± 12.9	54.1 ± 16.1	0.3
Posttest	67.6 ± 6.6	55.3± 5.8	< 0.0001 ^c^
Paired t test	< 0.0001 ^c^	0.07	-
**General Health**			
Pretest	51.2 ± 23.2	52.9 ± 20.6	0.90
Posttest	75.8 ± 18.8	55 ± 21.8	< 0.0001 ^c^
Paired t test	< 0.0001 ^c^	0.07	-
**Social Functioning**			
Pretest	64.8 ± 19.6	63.8 ± 20.8	0.72
Posttest	91.2 ± 10.3	65.4 ± 21.1	< 0.0001 ^c^
Paired t test	< 0.0001 ^c^	0.08	-
**Mental Health**			
Pretest	53.3 ± 11.6	51.4 ± 17.9	0.16
Posttest	70.4 ± 5.8	52.4 ± 17.8	< 0.0001 ^c^
Paired t test	< 0.0001 ^c^	0.13	-
**Emotional Role**			
Pretest	47.4± 40.8	35.4 ± 42.3	0.05
Posttest	99 ± 10.3	40.4 ± 43.2	< 0.0001 ^c^
Paired t test	< 0.0001 ^c^	0.09	-

^a^ The attainable score is 0-100 in all Dimensions.

^b^ Data are presented as mean ± SD.

^c^ It is significant at α level less than 0.05.

**Table 4.** Mean scores of Social Support Dimensions in Intervention Group 2 (Member of Addicts Family), Before and After the Educational Intervention ^a, b^

	Values
**Personal**	
Pretest	12.7 ± 5.3
Posttest	24.9 ± 2.2
Paired t test	< 0.0001 ^c^
**Family support**	
Pretest	17.1 ± 6.2
Posttest	25.3 ± 2.4
Paired t test	< 0.0001 ^c^
**Friend support**	
Pretest	13.7 ± 5.6
Posttest	25.2 ± 2.7
Paired t test	< 0.0001 ^c^

^a^ The attainable score is 0-100 in all Dimensions.

^b^ Data are presented as mean ± SD.

^c^ It is significant at α level less than 0.05.

**Table 5.** Mean scores of Quality of Life Dimensions in Intervention Group 2 (Member of Addicts Family), Before and After the Educational Intervention ^a, b^

	Values
**Physical functioning**	
Pretest	80.7 ± 20.6
Posttest	97 ± 8.6
Paired t test	< 0.0001 ^c^
**Physical Role**	
Pretest	52.4 ± 35.6
Posttest	100 ± 0
Paired t test	< 0.0001 ^c^
**Body pain**	
Pretest	73.7 ± 23.1
Posttest	98.2 ± 6
Paired t test	< 0.0001 ^c^
**Vitality**	
Pretest	60.2 ± 18.8
Posttest	78 ± 0.1
Paired t test	< 0.0001 ^c^
**General health**	
Pretest	63.2 ± 27.5
Posttest	94.2 ± 12.9
Paired t test	< 0.0001 ^c^
**Social functioning**	
Pretest	73± 17.9
Posttest	96.3± 7.1
Paired t test	< 0.0001 ^c^
**Mental health**	
Pretest	58.4 ± 20.9
Posttest	79 ± 10.3
Paired t test	< 0.0001 ^c^
**Emotional Role**	
Pretest	48.1 ± 38.8
Posttest	100 ± 0
Paired t test	< 0.0001 ^c^

^a^ The attainable score is 0-100 in all Dimensions.

^b^ Data are presented as mean ± SD.

^c^ It is significant at α level of less than 0.05.

## 5. Discussion

As many experts pointed out, social support is a crucial component of health promotion interventions and its relation with QOL and health promoting behaviors has been investigated in various studies (29, 30). Social support is also an important factor for methamphetamine users (31). At therapy sessions, many methamphetamine users stated that family support, especially emotionally and financially are key factors in quitting and lack of support and family and/or society's prejudice are main triggers of relapse (32). The main goal of the present study was to evaluate effects of the family-centered empowerment model on social support and QOL of methamphetamine users and their families. Results of this study, are in line with other studies (33, 34) and showed that the family-centered empowerment model based on a supportive psychotherapy plan can affect all dimensions of social support and HRQOL of methamphetamine users and their families. In other words, it seems that educational interventions based on this model were well received and methamphetamine users and their families experienced favorable outcomes by adopting them which means that individuals could improve their social support and QOL by receiving educational interventions including: learning problem solving skills to deal with daily life issues properly, opportunities for emotional depletion, relaxation techniques and positive imagination to reduce anxiety and create inner peace, evaluation guilt and methods to overcome it, learning techniques to increase self confidence and self-esteem by emphasizing on individuals’ capabilities and encouragement to participate in everyday chores. In the present study, mean score of perceived social support dimensions including support from parents, close friends, relatives and friends in the two educational groups changed significantly after intervention which is consistent with the study by Heidari et al. on the effects of supportive psychotherapy sessions on relapse in drug abuse (35) and also consistent with a study by Knowlton et al. on the positive effect of teaching social support on intravenous drug users (36). Social support is a key element in drug withdrawal process. A study in the USA showed that methamphetamine users believed that defected parent-child relations, lack of social relations and no family support are related to suicide (37). It seems that receiving proper social support gets the individual eager to learn and practice coping strategies like problem solving, social skills and communication skills and care for his own health, gradually leading to an effective therapy (38) a finding which is in line with Hosseinian’s findings (39). Clearly this vulnerable group of patients (methamphetamine users) need supportive psychotherapy and education to heal their mental and physical wounds and enjoy a healthy and high quality life. There was also a positive and significant correlation between perceived social support index and all dimensions of HRQOL meaning that receiving more support from family or friends can improve QOL of methamphetamine users and their families; this finding is in line with other similar studies (14, 40, 41). Social support can be a very powerful and beneficial force in the recovery process and enhance an addict’s QOL and mental health (42, 43). A limitation to this study was that some patients could not focus on questions and answers due to concentration impairment in the early stages of methamphetamine withdrawal. Also although cultural diversity was considered in the present study, generalization to different cultures is limited. Overall, the present findings suggest that family-centered empowerment model which is easily adapted to meth users and their families leads to improved social support and QOL. Therefore, placing an emphasis on family-centered strategies contributes to health promotion of methamphetamine users and thus, their family and society.
